# Efficacy of dietary odd-chain saturated fatty acid pentadecanoic acid parallels broad associated health benefits in humans: could it be essential?

**DOI:** 10.1038/s41598-020-64960-y

**Published:** 2020-05-18

**Authors:** Stephanie Venn-Watson, Richard Lumpkin, Edward A. Dennis

**Affiliations:** 1 Seraphina Therapeutics, Inc., San Diego, CA 92106 USA; 2 Epitracker Inc., San Diego, CA 92106 USA; 3 Department of Chemistry and Biochemistry and Department of Pharmacology, University of California at San Diego, La Jolla, CA 92093-0601 USA

**Keywords:** Cardiovascular diseases, Endocrine system and metabolic diseases, Nutrition disorders, Dyslipidaemias, Metabolic syndrome, Non-alcoholic fatty liver disease, Non-alcoholic steatohepatitis, Risk factors, Anaemia

## Abstract

Dietary odd-chain saturated fatty acids (OCFAs) are present in trace levels in dairy fat and some fish and plants. Higher circulating concentrations of OCFAs, pentadecanoic acid (C15:0) and heptadecanoic acid (C17:0), are associated with lower risks of cardiometabolic diseases, and higher dietary intake of OCFAs is associated with lower mortality. Population-wide circulating OCFA levels, however, have been declining over recent years. Here, we show C15:0 as an active dietary fatty acid that attenuates inflammation, anemia, dyslipidemia, and fibrosis *in vivo*, potentially by binding to key metabolic regulators and repairing mitochondrial function. This is the first demonstration of C15:0’s direct role in attenuating multiple comorbidities using relevant physiological mechanisms at established circulating concentrations. Pairing our findings with evidence that (1) C15:0 is not readily made endogenously, (2) lower C15:0 dietary intake and blood concentrations are associated with higher mortality and a poorer physiological state, and (3) C15:0 has demonstrated activities and efficacy that parallel associated health benefits in humans, we propose C15:0 as a potential essential fatty acid. Further studies are needed to evaluate the potential impact of decades of reduced intake of OCFA-containing foods as contributors to C15:0 deficiencies and susceptibilities to chronic disease.

## Introduction

Over the past 40 years, diets with lower saturated fats have been recommended to decrease cholesterol levels and subsequent risks of heart disease^1^. In the U.S., during the 20-year period following this initial recommendation, the average individual’s daily intake of whole fat milk was reduced more than 4-fold, from 283 to 65 grams per day, in an effort to lower dietary saturated fats^2,3^. Despite this drop in whole fat milk intake, the global prevalence of obesity, type 2 diabetes, metabolic syndrome, and nonalcoholic fatty liver disease has increased^4–6^. Further, an 18-year longitudinal study including over 25,000 individuals demonstrated that children fed whole fat milk had a lower risk of having obesity compared to children who were provided fat-free or 1% fat milk, and multiple studies have demonstrated associations between higher dietary intake of full-fat dairy and reduced risk of type 2 diabetes and cardiovascular disease^7–9^. As such, there is a need re-evaluate potential health risks versus benefits of dietary dairy fats^10^.

Among fatty acids present in whole fat milk, approximately 68% are even-chain saturated fatty acids (ECFAs), including myristic acid (C14:0), palmitic acid (C16:0) and stearic acid (C18:0)^11^. In contrast, odd-chain saturated fatty acids (OCFAs), pentadecanoic acid (C15:0) and heptadecanoic acid (C17:0), represent only 1% and 0.5%, respectively, of fatty acids in whole fat milk^11^. While dietary ECFAs have been associated with increased risk of inflammation, heart disease, and type 2 diabetes in humans^12–15^, higher dietary intake and circulating levels of OCFAs have been associated with lower risks of adiposity, chronic inflammation, cardiovascular disease, metabolic syndrome, type 2 diabetes, nonalcoholic steatohepatitis (NASH), chronic obstructive pulmonary disease, pancreatic cancer and other conditions^14–27^. In a prospective cohort involving over 14,000 people followed for an average of 14 years, increased dietary intake of OCFAs was associated with lower mortality in both men and women, while higher ECFA intake was associated with higher mortality in women^28^. Current dietary saturated fat recommendations do not discriminate between ECFAs and OCFAs, and the growing body of literature demonstrating opposing associations of ECFAs and OCFAs with disease and health have led to calls to refine global saturated fatty acid dietary guidelines^29,30^. This may be particularly important since population-wide concentrations of circulating OCFAs are decreasing^31^. While OCFAs have been routinely measured as biomarkers of dairy fat intake, and epidemiological studies provide evidence of associations between OCFAs and better health^32^, there has been a paucity of controlled studies to evaluate OCFAs as active dietary fatty acids that directly confer the health benefits to which they are associated.

Bottlenose dolphins (*Tursiops truncatus*) are long-lived, large-brained mammals that can naturally develop metabolic syndrome and NASH^33,34^. We previously reported that, similar to humans, dolphins with higher serum OCFAs have a lower risk of metabolic syndrome and liver disease^35^. In an effort to continually improve dolphin health and to understand how changes in the ocean may impact wild dolphins’ dietary needs, a group of dolphins was provided a modified fish diet higher in OCFAs. The modified diet effectively increased serum C15:0 and C17:0 concentrations, and components of metabolic syndrome normalized within three months^35^. This pilot study supported that increased dietary OCFAs may improve metabolic health. Further, our study showed that beyond dairy products, dietary OCFA intake can be increased using specific types of fish. This is consistent with previous studies documenting measurable OCFAs outside of dairy products, including some types of fish, plants, and bacteria^36–39^. Because our study used modified fish diets and not pure OCFAs, however, we were not able to conclude that increased OCFAs directly caused the observed health benefits.

Due to the need for robust, controlled studies to evaluate potential direct activities of OCFAs, we conducted a series of *in vitro* and *in vivo* studies with pure (99%) OCFAs to assess their potential causative roles in safely attenuating targeted chronic comorbidities. Herein, given the known role of omega-3 and other dietary fatty acids as natural ligands for the body’s orchestrators of metabolism (called peroxisome proliferator-activated receptors, or PPARs) that may protect against insulin resistance, type 2 diabetes, inflammation and NASH, we tested OCFAs for PPAR agonist activities^40–43^. Due to potential roles of fatty acids on mitochondrial function and reactive oxygen species (ROS) that can, in turn, increase the risk of cardiometabolic diseases, we evaluated impacts of OCFAs on mitochondrial function and ROS production^44^. OCFAs were tested across a variety of human cell systems mimicking chronic inflammatory and fibrotic disease states. The effects of OCFA oral supplementation were then studied in *in vivo* models of cardiometabolic, inflammatory, liver, hematologic, and fibrotic diseases. Based on our findings, we reviewed current scientific literature related to OCFAs, with a lens on potential support for OCFAs as essential dietary fatty acids.

## Results

### C15:0 is a dual, partial PPAR α/δ agonist

Peroxisome proliferator-activated receptors (PPARs) are present throughout the body and help to regulate metabolism and inflammation by detecting and responding to dietary fats^40^. Here, OCFAs C15:0 and C17:0, as well as ECFAs C14:0 and C16:0, were evaluated at 10 concentrations for cell-based PPAR agonist activities, including all three PPAR isoforms, α, δ, and γ. C15:0 was a dual, partial PPARα and PPARδ agonist, with maximum activities of 65.8% and 52.8%, respectively, compared to that of positive controls (GW7647 and L-165,041) (Supplement Fig. 1). Effective concentrations of C15:0 needed to reach half-maximum activities for PPARα and PPARδ were 11.5 and 2.7 µM, respectively. In comparison to C15:0, C17:0 had lower maximum PPARδ agonist activities (39.8%), required higher concentrations to achieve half-maximum PPARδ activities (17.4 µM), and was not a PPARα agonist up to 100 µM (Supplement Fig. 1). Dual PPARα/δ agonist activities similar to C15:0 were present for C14:0 and C16:0, suggesting that carbon chain length may be a determinant of PPARα/δ binding. No saturated fatty acids had PPARγ agonist activities at concentrations less than 100 µM.

### C15:0 had no off-target pharmacological activities

Due to detected dual, partial PPARα/δ agonist activities, C15:0 was further tested at 10 concentrations for other agonist and antagonist activities across 76 assays and 47 genes and targets that are relevant to compound safety and potent pharmacological mechanisms of action, including G-protein coupled receptors, kinases, transporters, ion channels, nuclear receptors, and non-kinase enzymes. No off-target activities were detected with C15:0 when tested up to 20 µM (Suppl Table 1).

### C15:0 repaired mitochondrial function

**Figure 1:**
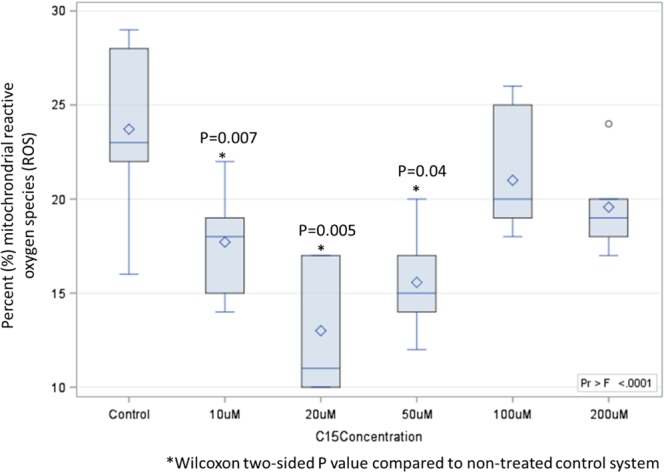
Reduced mitochondrial reactive oxygen species production in stressed human cell systems supplemented with C15:0 at 10, 20 and 50 µM compared to non-supplemented controls. Endpoint of upper whisker, maximum; upper edge of box, 75^th^ percentile; line inside box, median; diamond, mean; lower edge of box, 25^th^ percentile; endpoint of lower whisker, minimum; circle, outlier.

Mitochondrial dysfunction, including increased production of reactive oxygen species (ROS), is a key component of the degradative process of aging and numerous chronic diseases, including cardiometabolic diseases^44^. Since fatty acids can affect mitochondrial function^45^, the effects of OCFAs and ECFAs on repairing mitochondrial function and reducing mitochondrial ROS were evaluated in serum starved HepG2 cells. Here, C15:0 had a dose-response u-curve effect on mitochondrial function, including lower mitochondrial ROS production in cell systems supplemented at 10 µM (17.8 ± 2.7%, P = 0.04), 20 µM (12.9 ± 3.2%, P = 0.005) and 50 µM (15.4 ± 2.6%, P = 0.007) compared to non-supplemented control cell systems (23.4 ± 4.3%) (Fig. 1). There were no differences in ROS production when comparing cells supplemented at higher C15:0 concentrations (100 and 200 µM) compared to non-supplemented controls. Among a variety of other OCFAs and ECFAs evaluated (C13:0 through C18:0), C15:0 through C18:0 (20 µM) had lower mitochondrial ROS compared to the non-supplemented control group, while C13:0 and C14:0 did not significantly lower mitochondrial ROS (Suppl Fig. 2).

### C15:0 was non-cytotoxic across multiple primary human cell lines

Safety of C15:0 was further assessed by exposing 12 primary human cell systems with venular endothelial cells, peripheral blood mononuclear cells, B cells, bronchial epithelial cells, dermal fibroblasts, coronary artery smooth muscle cells, keratinocytes, lung fibroblasts or macrophages, at multiple concentrations (0.7, 2.2, 6.7 and 20 µM). Based on a definition of cytotoxicity in which more than 50% of total protein in the cell system was reduced, C15:0 did not induce cytotoxicity in any of the 12 cell systems (Suppl Table 2).

### C15:0 reduced proinflammatory and profibrotic states in human cell systems

**Figure 2:**
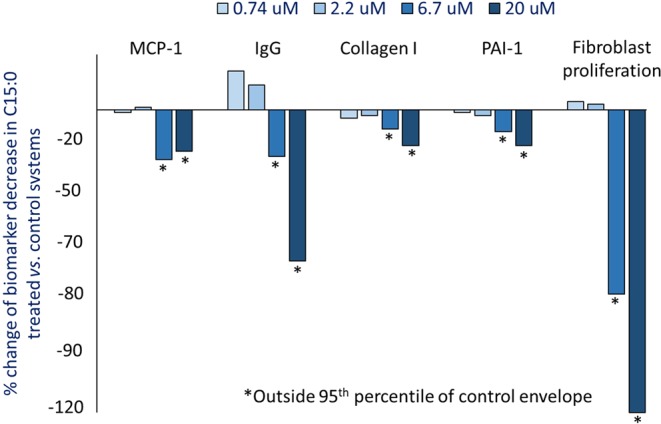
Annotated dose-dependent anti-inflammatory and antifibrotic activities of C15:0 (0.74, 2.2, 6.7 and 20 µM) in primary human cell systems mimicking inflammation and fibrosis. MCP-1 = monocyte chemoattractant protein 1, sIgG = secreted immunoglobulin G, PAI-1 = plasminogen activation inhibitor 1.

**Figure 3:**
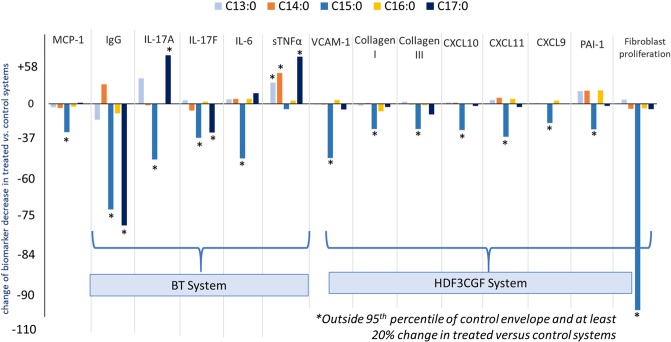
Annotated dose-dependent anti-inflammatory and antifibrotic activities of saturated fatty acids (C13:0, C14:0, C15:0, and C16:0 in 20 µM) in primary human cell systems mimicking inflammation and fibrosis. MCP-1 = monocyte chemoattractant protein 1; IgG = immunoglobulin G; IL-17A, IL-17F, IL-6 = interleukin 17 A, 17 F and 6; sTNFα = secreted tumor necrosis factor alpha; VCAM-1=vascular adhesion molecule 1, CXCL10, CXLX11, CXCL9 = chemokine ligand 10, 11 and 9; PAI-1 = plasminogen activation inhibitor 1). BT System = Primary human cell system in Eurofins/DiscoverX BioMAP Diversity PLUS panel modeling T cell dependent B cell proliferation; HDF3CGF System = Primary human cell system in Eurofins/DiscoverX BioMAP Diversity PLUS panel modeling matrix/tissue remodeling in the context of Th1-type inflammation, including fibrosis and chronic inflammation.

Due to opposing associations of OCFAs and ECFAs with better *versus* worse cardiometabolic and liver health, respectively, in human populations, OCFAs (C13:0, C15:0, and C17:0) and ECFAS (C14:0 and C16:0) were tested for anti-inflammatory and antifibrotic activities, including measuring 148 biomarker readouts across 12 primary human cell-based systems mimicking inflammation and fibrosis. C15:0 was tested at four concentrations (0.7, 2.2, 6.7 and 20 µM), and the remaining saturated fatty acids were tested at 20 µM. Here, C15:0 had dose-dependent, annotated anti-inflammatory activities, including reduced monocyte chemoattractant protein 1 (MCP-1) and secreted immunoglobulin G (IgG) (Fig. 2). C15:0 also had antifibrotic activities, including reduced Collagen I, plasminogen activation inhibitor 1 (PAI-1), and 72-hour fibroblast proliferation (Fig. 2). Anti-inflammatory and antifibrotic activities were present at both 6.7 and 20 µM. C15:0 cell-based anti-inflammatory and antifibrotic activities at 20 µM were better than C17:0 at the same concentration; other saturated fatty acids (C13:0, C14:0 and C16:0) had no anti-inflammatory or antifibrotic activities (Fig. 3). Because C14:0, C15:0, and C16:0 all had similar dual PPARα/δ agonist activities (reported above), results from our human cell phenotypic screening support that C15:0 activities go beyond C15:0's role as a natural PPARα/δ fatty acid ligand. This study also supports that a relatively minor increase in C15:0 concentrations (e.g. from 2.2 µM to 6.7 µM) can positively impact its anti-inflammatory and antifibrotic activities.

### Oral C15:0 achieved active concentrations *in vivo*

**Figure 4:**
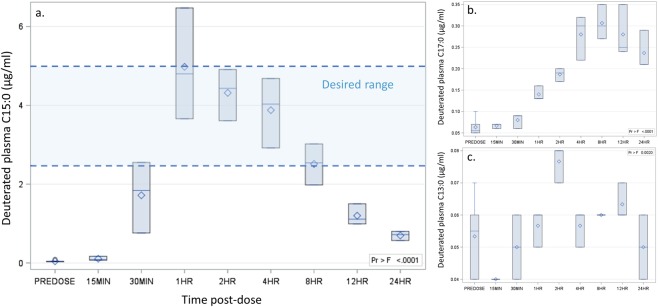
Plasma deuterated C15:0 (**a**), C17:0 (**b**), and C13:0 (**c**) concentrations achieved over 24 h in male Sprague Dawley rats (n = 6 total, 3 per time point between 15 min and 12 h) dosed orally once with deuterated C15:0 (35 mg/kg body weight). Upper edge of box, maximum; line inside box, median; diamond, mean; lower edge of box, minimum.

Given that C15:0 demonstrated cell-based anti-inflammatory and antifibrotic activities between 6.7 and 20 µM (equal to 2.5 to 5 µg/ml), we first sought to understand oral doses of C15:0 needed to achieve these plasma concentrations in appropriate models. Here, 8- to 10-week old Sprague Dawley rats (n = 6 males) dosed orally once with C15:0 at 35 mg/kg body weight had increased C15:0 plasma levels within 30 minutes (Fig. 4). A maximum C15:0 plasma concentration of 4.98 µg/ml (20 µM) was achieved at 1 hour. C15:0 plasma levels were elevated above baseline levels throughout the 24-hour period, with a minimum concentration of 0.7 µg/ml. Thus, a single oral dose of C15:0 at 35 mg/kg succeeded in achieving our targeted active plasma concentrations in this rodent model, between 2.5 to 5 µg/ml (equivalent to 6.7 to 20 µM), from 1 to 8 hours post-dose. Plasma total C17:0 levels also increased, albeit less so than C15:0, following a single oral dose of C15:0; similar, sustained increases were not apparent with C13:0 (Fig. 4). These findings support *de novo* elongation of C15:0 to C17:0.

### Daily oral C15:0 maintained safety *in vivo* at high doses over 14 days

To further evaluate the safety of C15:0 at increasing doses, Sprague Dawley rats (n = 10 per group, 5 females and 5 males, 7 to 8 weeks old) were dosed orally once daily for 14 days with C15:0 at 35, 175 and 350 mg/kg body weight. A non-dosed vehicle control group was included. Safety assessments included clinical observations, body weight, food intake, clinical chemistries, and histology (liver, kidney, heart, and adrenal glands). Additionally, total plasma C15:0 and C17:0 concentrations were measured at Day 14. There were no mortalities or observed abnormal behaviors in animals throughout the 14-day study across all study groups, and there were no significant differences when comparing body weights and organ weight-to-body weight ratios or the prevalence of abnormal clinical chemistry values or histologic observations between C15:0-supplemented and non-supplemented control animals (Suppl Table 3). Following 14 days of daily oral supplementation with C15:0 at three increasing doses, increasing plasma total C15:0 and total C17:0 concentrations were evident (p < 0.0001) (Suppl Fig. 3). Males dosed 35, 175, or 350 mg/kg oral C15:0 had significantly higher total C15:0 and C17:0 plasma concentrations at Day 14 compared to baseline controls (Suppl Table 3). Females dosed 175 or 350 mg/kg oral C15:0 had significantly higher total C15:0 plasma concentrations at Day 14 compared to baseline controls, while only females dosed 350 mg/kg oral C15:0 had significantly higher C17:0 plasma concentrations compared to baseline controls (Suppl Table 3).

### Low-dose daily oral C15:0 attenuated a pro-inflammatory state and lowered glucose and cholesterol *in vivo*

**Figure 5:**
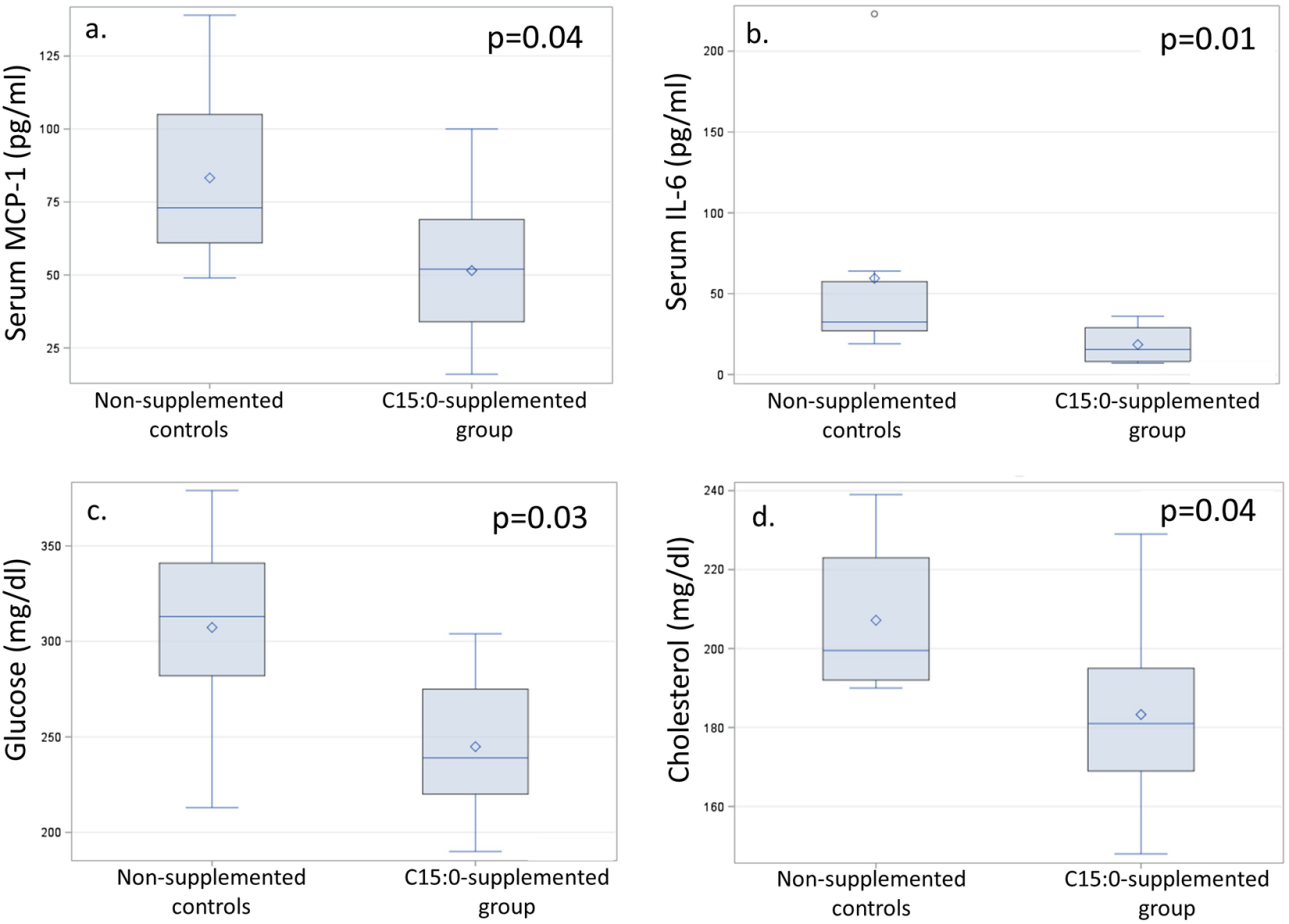
Comparisons of circulating concentrations of the pro-inflammatory chemokine, monocyte chemoattractant 1 (MCP-1) (**a**), pro-inflammatory cytokine, interleukin 6 (IL-6) (**b**), glucose (**c**), and total cholesterol (**d**) between high fat diet-induced obese *in vivo* model supplemented with low dose daily oral C15:0 (pentadecanoic acid, 5 mg/kg) over 12 weeks and non-supplemented controls. Wilcoxon two-sided p values.

We assessed the effects of daily oral C15:0 (low dose, 5 mg/kg) and daily oral C17:0 (low and high dose, 5 and 50 mg/kg) on inflammation and cardiometabolic conditions in obese C57BL/6 J mice (n = 10 males per group) fed a high-fat diet (60% kilocalories fat). Mice supplemented with oral C15:0 for 90 days at low doses (5 mg/kg) had lower circulating levels of the proinflammatory chemokine, monocyte chemoattractant protein 1 (MCP-1), and the proinflammatory cytokine, interleukin 6 (IL-6) compared to non-supplemented controls (Fig. 5). The C15:0-supplemented group also had lower glucose, lower cholesterol, and lower percent body weight gain on the high fat diet compared to non-supplemented controls (Fig. 5, Suppl Table 4). In contrast, mice supplemented with daily low dose C17:0 (5 mg/kg) had no significant differences in clinical chemistry values compared to non-supplemented, diseased controls, while high dose C17:0 (50 mg/kg) supplemented mice had lower serum MCP-1 compared to controls (Suppl Table 4). Mice supplemented with low dose oral C15:0 had higher serum concentrations of both lysophosphatidylethanolamine (LPE) C15:0 and LPE C17:0 compared to non-supplemented controls (Suppl Table 5, Suppl Fig. 4).

### Daily oral C15:0 attenuated anemia, inflammation, liver iron overload and NASH *in vivo*

**Figure 6:**
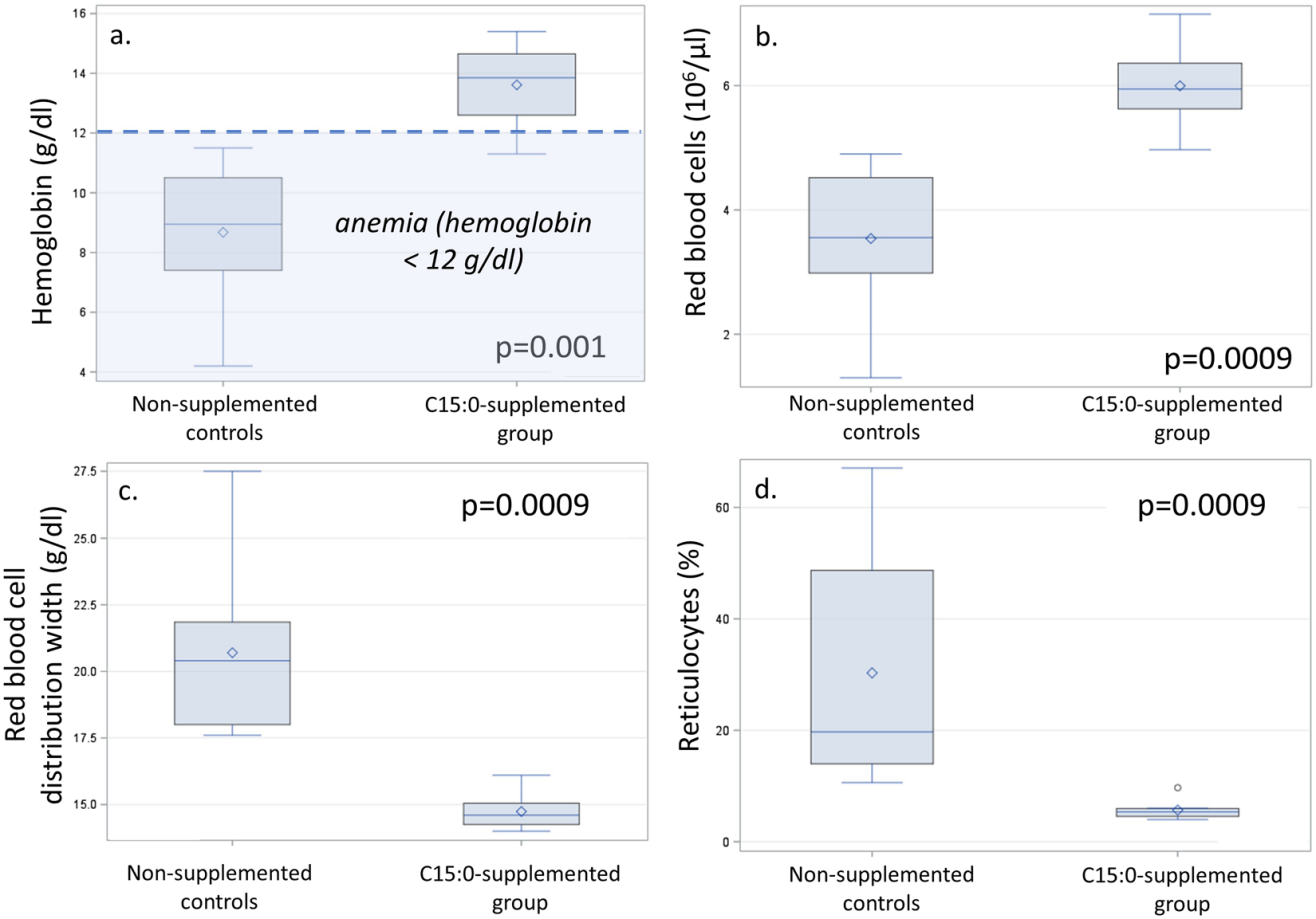
Comparisons of red blood cell indices, including hemoglobin (**a**), red blood cell counts (**b**), red blood cell distribution width (**c**), and reticulocytes (**d**) between high fat-diet fed *in vivo* models for nonalcoholic steatohepatitis (NASH) supplemented with higher doses of daily oral C15:0 (pentadecanoic acid, 35 mg/kg) over 11 weeks and non-supplemented controls. Wilcoxon two-sided p values.

**Figure 7:**
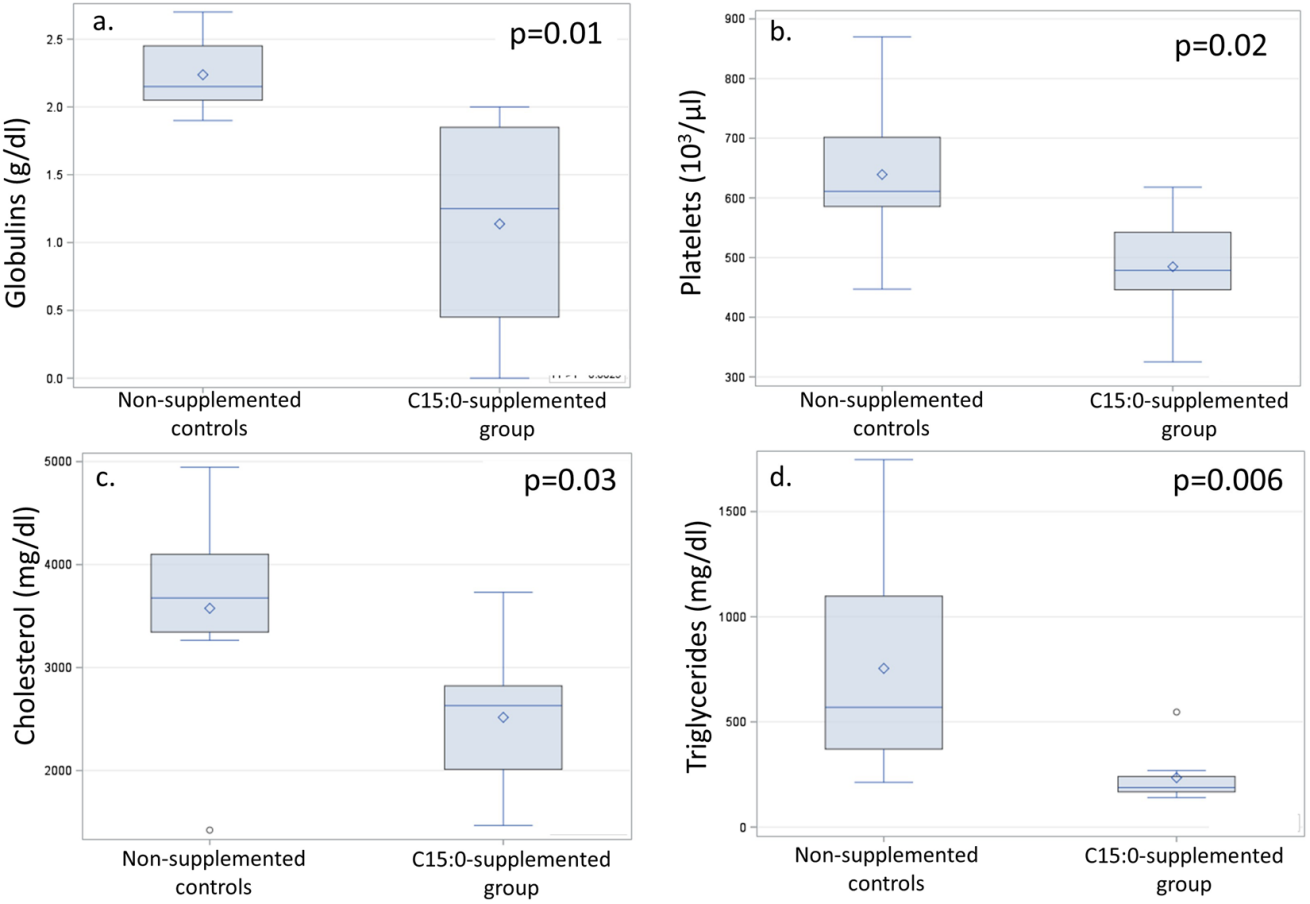
Comparisons of circulating globulins (**a**), platelets (**b**), cholesterol (**c**), and triglycerides (**d**) between high fat-diet fed *in vivo* models for nonalcoholic steatohepatitis (NASH) supplemented with higher doses of daily oral C15:0 (pentadecanoic acid, 35 mg/kg) over 11 weeks and non-supplemented controls. Wilcoxon two-sided p values.

New Zealand white rabbits fed a high-fat, high-cholesterol diet (4% peanut oil and 0.5% cholesterol) developed hypercholesterolemia, anemia, and NASH^46^. After 2 weeks on this diet, animals were supplemented with daily oral C15:0 (35 mg/kg body weight) for 11 weeks and compared to both non-supplemented diseased controls and standard diet healthy controls (n = 8 males per group). Daily, oral C15:0 supplementation attenuated a regenerative hemolytic anemia induced by the high fat, high cholesterol diet (Fig. 6, Suppl Table 6). Specifically, C15:0 supplementation raised hemoglobin, hematocrit, and red blood cell count, and lowered nucleated red blood cells, red blood cell distribution width, and reticulocytes. In this model, these changes are consistent with decreased loss of fragile red blood cells and lowered need for new red blood cell production^46^. Further, C15:0-supplemented animals had lower cholesterol, triglycerides, globulins, and platelets compared to non-supplemented diseased controls (Fig. 7). Additionally, multiple liver health indices in C15:0-supplemented animals, including bilirubin and icterus were lower than non-supplemented diseased controls, matching that of healthy controls (Supplement Table 6). Histologically, C15:0-supplemented animals also had less severe liver fibrosis and liver iron staining scores within Kupffer cells compared to non-supplemented diseased controls. Unlike the non-supplemented diseased controls, C15:0-supplemented animals did not progress from Stage 2 to Stage 3 (bridging) fibrosis (Suppl Table 6).

### There is supportive evidence of C15:0 as a potential essential fatty acid

Essential fatty acids are defined as active dietary fatty acids that: (1) are required to maintain a healthy physiological state, (2) are not made at adequate levels endogenously, and (3) require dietary intake in order to maintain healthy concentrations in the body^47^. Given our demonstration of C15:0 and C17:0 as active dietary fatty acids, we reviewed the literature for evidence supporting or negating C15:0 and C17:0 as potential essential fatty acids (Table 1). Due to reported direct correlations between dietary C15:0 intake and circulating C15:0 concentrations, indicative of primarily diet-based drivers of circulating C15:0, and evidence of endogenous production of C17:0, only C15:0 had supportive evidence across all three criteria that were consistent with a potential essential fatty acid.

**Table 1:** Summary of evidence supporting or refuting odd-chain saturated fatty acids (pentadecanoic acid, C15:0 and heptadecanoic acid, C17:0) as potential essential fatty acids based on key criteria.

Essential fatty acid criterium	Evidence	References
*Is it a dietary fatty acid?*		
Pentadecanoic acid (C15:0) *Yes*	**Strong**. C15:0 is well established as an odd-chain saturated fatty acid present in foods, especially dairy fat, and has been used as a reliable biomarker of dairy fat intake.	^11,32,36,37,57^
Heptadecanoic acid (C17:0) *Yes*	**Strong**. C17:0 is well established as an odd-chain saturated fatty acid present in foods, especially dairy fat.	^11,36,37^
*It is an active fatty acid?*		
C15:0 *Yes*	**Strong**. Evidence provided herein supports C15:0 as: (1) a partial peroxisome proliferator-activated receptor alpha/delta (PPARα/δ) ligand and agonist, (2) having dose-dependent mitochondrial reparative functions, (3) having dose-dependent anti-inflammatory and anti-fibrotic properties in human cell lines, (4) a stabilizer of red blood cells *in vivo*, and (5) a means to attenuate pro-inflammatory states, inflammation, anemia, liver iron overload, and liver fibrosis and lowering cholesterol and glucose *in vivo*. Additionally, OCFAs have been demonstrated to protect HepG-2 cells from palmitate-induced insulin resistance via PPARα agonist activity.	New studies detailed in current article +^78^
C17:0 *Yes*	**Strong**. Evidence provided herein supports C17:0 as: (1) a partial peroxisome proliferator-activated receptor delta (PPARδ) ligand and agonist, (2) having mitochondrial reparative functions, and (3) reducing a pro-inflammatory state in primary human cell lines and *in vivo*. Additionally, OCFAs have been demonstrated to protect HepG-2 cells from palmitate-induced insulin resistance via PPARα agonist activity.	New studies detailed in current article +^78^
*Is it required to maintain a healthy physiological state?*		
C15:0 *Possibly*	**Associative**. Numerous, large scale epidemiological studies repeatedly demonstrate associations between higher C15:0 dietary intake, higher circulating C15:0 concentrations, and lower risks of (1) mortality and (2) having or developing chronic conditions, including: inflammation, cardiovascular disease, type 2 diabetes, obesity, metabolic syndrome, non-alcoholic fatty liver disease/severe non-alcoholic steatohepatitis, and pancreatic cancer. As population-wide circulating concentrations of C15:0 have declined, the prevalence of these conditions has increased.	^4–6,14–28,31^
C17:0 *Possibly*	**Associative**. Numerous, large scale epidemiological studies repeatedly demonstrate associations between higher circulating C17:0 concentrations, and lower risks of having or developing chronic conditions, including: inflammation, cardiovascular disease, type 2 diabetes, obesity, metabolic syndrome, non-alcoholic fatty liver disease/severe non-alcoholic steatohepatitis, and pancreatic cancer. As population-wide circulating concentrations of C17:0 have declined, the prevalence of these conditions has increased.	^4–6,14–28,31^
*Do humans lack the ability to endogenously produce enough of this fatty acid to maintain adequate blood concentrations?*		
C15:0 *Yes*	**Moderate to Strong**. Multiple studies demonstrate linear correlations between the amount of C15:0 ingested and C15:0 concentrations in the blood and adipose tissue, supporting that C15:0 blood levels are driven primarily by C15:0 dietary intake and not endogenous production. In one study, circulating C15:0 concentrations were not influenced by gut microbiota. Declines in population circulating C15:0 concentrations over recent years appears to be attributed to lowered intake of foods containing C15:0, including whole dairy fat. While higher circulating C15:0 levels are associated with better health (see above), definitions of ‘adequate’ have not been confirmed.	^31,32,57,74^
C17:0 *No*	**Strong**. Studies demonstrate a lack of correlation between the amount of C17:0 ingested and circulating C17:0 concentrations, supporting that C17:0 blood levels are influenced by endogenous production.	^32,57,73^

## Discussion

Our series of studies herein support that dietary odd-chain saturated fatty acids (OCFAs), especially C15:0, are active fatty acids having cell-based activities and *in vivo* efficacy that align with health benefits, including lower risk of inflammation, cardiometabolic diseases, and NASH, to which they have been associated in numerous large epidemiological studies in humans^14–29^.

Chronic low-grade inflammation, driven by proinflammatory chemokines and cytokines, contributes to cardiometabolic comorbidities and the aging process^48–50^. Here, daily oral supplementation with C15:0 and C17:0 lowered proinflammatory states in obese mice with metabolic syndrome, as well as lowered proinflammatory biomarkers in primary human cell systems mimicking chronic inflammation. C15:0 and C17:0 lowered monocyte chemoattractant protein 1 (MCP-1), a proinflammatory chemokine; C15:0 also lowered proinflammatory chemokines (CXCL9, 10 and 11), cytokines (IL-6, -17A), and adipokines (platelet activation inhibitor 1, or PAI-1). MCP-1, which can be induced by the even-chain saturated fatty acid (ECFA), C16:0, contributes to chronic inflammation present in chronic cardiometabolic diseases, and elevated MCP-1 concentrations have also been associated with pancreatic cancer^51–53^. Further, circulating MCP-1 has been proposed as a measurement of biological age in mice and frailty in humans^54^. Our studies suggest that active lowering of proinflammatory states, including lowered MCP-1, by OCFAs may contribute to their associations with lower adipokines and lower risks of type 2 diabetes, cardiovascular disease, pancreatic cancer, and mortality^14,15,19,23,28^.

Dyslipidemia and hyperglycemia are components of metabolic syndrome, a cluster of conditions impacting approximately 1 in 3 people globally^55^. Metabolic syndrome increases the risk of type 2 diabetes, heart disease, and all-cause mortality. In our studies, daily oral C15:0 supplementation over 12 weeks lowered total cholesterol and glucose in an *in vivo* model with metabolic syndrome. Similarly, a case-cohort study involving 15,919 humans across eight European countries demonstrated associations between higher plasma phospholipid C15:0 and C17:0 concentrations and lower total cholesterol and triglycerides; conversely, higher ECFA concentrations were associated with higher triglycerides^56^. While higher circulating concentrations of both C15:0 and C17:0 have been associated with lower risks of type 2 diabetes, a controlled study in an animal model demonstrated that plasma increases of C17:0, and not C15:0, had linear correlations with decreased insulin and glucose^14,57^.

Our study data, paired with observational associations in human population studies, support that increased dietary and circulating C15:0 may have direct cholesterol- and triglyceride-lowering effects in humans. Further, our studies demonstrate that ingestion of C15:0 alone increases both C15:0 and C17:0 plasma concentrations *in vivo*, suggesting that ingestion of C15:0 may offer benefits of both raised circulating C15:0 and C17:0, including improved glucose control. Elongation of C15:0 to C17:0 has been demonstrated in MCF7 human cells transfected with human elongases encoded by elongation of very long-chain fatty acid 6 (ELOVL6), as well as ELOVL7, providing a feasible mechanism for increased C17:0 concentrations following C15:0 ingestion^58^.

Nonalcoholic steatohepatitis (NASH) is an advanced stage of nonalcoholic fatty liver disease, which is present in approximately 25% of the global population and involves fat deposition in the liver paired with inflammation and fibrosis^59^. There is currently has no cure for NASH, which is soon become the leading cause of hepatocellular carcinoma and end-stage liver failure globally^59^. Here, in addition to lowering cholesterol and triglycerides, C15:0 supplementation attenuated anemia, liver iron deposition, and severity of liver fibrosis in our *in vivo* NASH model. The NASH phenotype in our model specifically mimics anemia of inflammation (also called anemia of chronic disease) involving fragile red blood cells and associated dysmetabolic iron overload syndrome with severe liver fibrosis, which are risk factors for more severe comorbidities, including cardiovascular diseases and NASH in humans^46,60–63^. Consistent with our *in vivo* study, we also demonstrated that C15:0 causes a dose-dependent reduction in fibroblast proliferation, collagen I and III, and proinflammatory PAI-1 in primary human cell systems mimicking fibrotic diseases. Aligned with our findings, higher circulating C15:0 concentrations have been previously associated with lower liver enzymes and lower risks of NAFLD and severe NASH in humans^26,56,64^. Our study provides evidence that orally administered C15:0 can improve red blood cell stability and attenuate anemia, liver iron deposition and liver fibrosis that could prevent or decrease the severity of NASH.

Aging is a degradative process involving decreased cellular stability (i.e. cellular senescence), increased reactive oxygen species, mitochondrial dysfunction and chronic inflammation that contribute to the onset and severity of comorbidities of aging, including cardiovascular disease, type 2 diabetes and chronic anemia^65^. Here, in addition to reducing a proinflammatory state and improving red blood cell stability to attenuate anemia *in vivo*, our studies demonstrate the ability for C15:0 to reduce reactive oxygen species and repair mitochondrial function in human cells. Similar effects were observed with C17:0. These results are consistent with previously proposed mitochondrial-protective effects of OCFAs due to increased downstream production of succinate, which, in turn, can rescue mitochondrial function via mitochondrial complex II^66^. Due to the demonstrated ability for OCFAs to attenuate multiple components of aging, there is a need to better understand how OCFAs may have a causative role in observed reduced mortalities among people who ingest higher dietary OCFAs compared to those who have lower OCFAs in their diets^28^.

Essential fatty acids are defined as active dietary lipids not produced in high enough quantities *de novo* to maintain a healthy physiological state^47,67^. As components of the cell membrane lipid bilayer, essential fatty acids, and fatty acids in general, play important roles in cellular stability, cell signaling, and immune function^47,68^. Alpha-linolenic acid and linoleic acid have long been considered essential fatty acids, and recent studies have demonstrated that their downstream polyunsaturated fatty acid derivatives, arachidonic acid and docosahexaenoic acid, can alone support healthy development in mice^69,70^. Our studies demonstrate C15:0 and C17:0 as active dietary fatty acids that cause cell-based activities and *in vivo* efficacy paralleling previously established associations with lower risks of numerous chronic inflammatory, cardiometabolic and liver conditions in humans. Further, C15:0 and C17:0 attenuated key hallmarks of aging, which may help explain why higher dietary OCFA intake has been associated with lower mortality^28^. These outcomes led to our review of the literature regarding the potential for OCFAs to be essential fatty acids.

First, there is strong evidence supporting that circulating C15:0 concentrations are primarily driven by exogenous, diet-based sources, while C17:0 is readily produced *de novo*. Multiple studies have demonstrated that dietary C15:0 intake and associated circulating concentrations are linearly correlated^32,57,71^. This supports that, although C15:0 can be elongated from propionate or C13:0, there does not appear to be strong endogenous, *de novo* production of C15:0^58,72^. In addition to linear correlations between dietary and circulating C15:0 concentrations, evidence of C15:0 as a primarily exogenous fatty acid includes lack of peroxisomal 2-hydroxyacyl-CoA lyase and liver-based α-oxidation involvement in endogenous production of C15:0, lack of strong correlations between orally administered propionate with plasma C15:0 concentrations, and no evidence of effects of changing gut microbiota on circulating C15:0^57,72,73^. In contrast, C17:0 appears to be readily produced *de novo* via propionate, liver-based α-oxidation, and elongation of C15:0 via ELOVL6^58,72,73^. Combining these publications with our studies, we propose dietary C15:0 as an important diet-derived building block for C17:0, in part by elongation of C15:0 to C17:0 *via* ELOVL6; this hypothesis is further supported by *in vivo* dose response studies demonstrating a linear correlation between dietary and serum C15:0 concentrations and near-doubling of serum C17:0 concentrations over what would be expected given actual C17:0 consumed^57^.

Second, there is moderate to strong evidence that ‘adequate’ circulating C15:0 concentrations can be defined. A human population pharmacokinetic model, based on a series of studies involving oral administration of pure C15:0 to healthy adults and children, estimated that the average baseline circulating C15:0 concentration in people is 24.9 µM^74^. These levels are consistent with our studies, which demonstrated cell-based PPARα/δ agonist, anti-inflammatory, antifibrotic, and mitochondrial protective C15:0 activities between 10 and 50 µM, with most of our studies demonstrating optimal activities at 20 µM. Human pharmacokinetic studies support that a single dose of 200 mg of C15:0 results in 20 µM circulating C15:0 concentrations (approximately 5 µg/ml)^75^. Further, the average reported dietary intake of C15:0, based on a population of women from the 1990s, was 220 ± 100 mg per day, which is consistent with the amount needed to achieve approximately 20 µM circulating C15:0 concentrations^76^. Combined, these studies support potential targeted circulating C15:0 concentrations from 20 to 30 µM. Further studies are needed to determine whether these concentrations may be defined as ‘adequate’ to help sustain physiological health.

Third, there is moderate supporting evidence that adequate circulating OCFA concentrations are needed to maintain physiological health. Large-scale population studies conducted globally repeatedly demonstrate that higher circulating concentrations of OCFAs (C15:0 and C17:0) are associated with lower risks of mortality, as well as having or developing obesity and numerous cardiometabolic and liver diseases^14–28^. These studies, however, are associative and do not establish causality. Our studies demonstrate OCFAs as active dietary fatty acids that directly attenuate multiple hallmarks of aging, as well as cardiometabolic, liver and inflammatory conditions, at concentrations consistent with circulating concentrations in humans, supporting a causal role of OCFAs in maintaining cardiometabolic, liver, and immune health. Our reliance on dietary C15:0 and maintained healthy circulating C15:0 concentrations is further supported by observed decreasing serum C15:0 concentrations in human populations over a recent 13-year period that are concurrent to the increasing prevalence of obesity, type 2 diabetes and NASH^4–6,31^. These studies support that targeted OCFA concentrations may be needed to maintain metabolic health, but more studies are needed to thoroughly evaluate the potential for OCFA deficiencies and associated deficiency disorders.

Our studies had several limitations. While the 24-hour and 14-day *in vivo* supplementation studies measured total plasma C15:0 and C17:0, they did not measure OCFA deposition in other sources, including red blood cells, lymph, and adipose tissue, where OCFAs can accumulate^32,35,77^. Further, while our mouse efficacy study included measurements of various serum lipid species containing C15:0 and C17:0, our efficacy studies lacked total circulating C15:0 and C17:0 concentrations, limiting our ability to confirm actual total circulating OCFA concentrations. Our pharmacokinetic studies, however, paired with previously published studies conducted with orally administered OCFAs using a variety of mammalian models with deuterated and C^14^-labeled C15:0, repeatedly confirm that orally administered OCFAs are bioavailable^32,57,74,77^. Because our efficacy studies were limited to males, we were unable to evaluate potential differences in OCFA efficacy based on sex. Thus, future studies with OCFAs would benefit from evaluating total OCFA concentrations in serum or plasma, red blood cells, lymph, and adipose tissues, and should include both males and females. It is worthy to note that our efficacy studies used diets containing relatively higher percentages of fat, especially saturated fats, with or without higher cholesterol compared to baseline diets to induce illness; given that our study design used dietary odd-chain saturated fatty acids to effectively attenuate illness in these models, more studies are needed to differentiate among various types of saturated fats and ‘high fat diets’ commonly used in *in vivo* cardiometabolic studies. While we propose C15:0 as a potential essential fatty acid, controlled studies feeding C15:0-depleted diets in appropriate models and evaluating them for impaired health could help confirm C15:0 as essential and define potential C15:0 deficiency syndromes.

In conclusion, our series of studies and review of the literature demonstrate that C15:0 (pentadecanoic acid), a trace dietary odd-chain saturated fatty acid present in dairy fat and some fish and plants: 1) is an active, exogenous fatty acid with anti-inflammatory, antifibrotic, red blood cell-stabilizing, and mitochondrial-reparative activities resulting in lowered cholesterol, triglycerides and glucose, and attenuated inflammation, anemia, and liver fibrosis *in vivo*, 2) may provide optimal health benefits against numerous cardiometabolic, liver and aging-associated conditions at circulating concentrations around 20 to 30 µM following ingestion of 200 to 300 mg C15:0 per day, and 3) may be needed in the diet to preserve a healthy metabolic state.

Based on these studies, we propose C15:0 as a potential essential fatty acid that may be needed to stem the global pandemics of obesity, cardiometabolic diseases and NASH. Further studies are needed to confirm this hypothesis, including assessing potential public health implications of population-wide declines in dietary whole dairy fat and other food-based sources of OCFAs over the past 40 years.

## Methods

### Animal welfare assurance

*In vivo* study protocols described herein were reviewed and approved by Institutional Animal Care and Use Committees at the facilities and were in accordance with the Final Rules of the Animal Welfare Act regulations (Code of Federal Regulations, Title 9), the Public Health Service Policy on Humane Care and Use of Laboratory Animals from the Office of Laboratory Animal Welfare, and the Guide for the Care and Use of Laboratory Animals from the National Research Council.

### Free fatty acids

All studies herein utilized synthetic free saturated fatty acids, including pentadecanoic acid, purchased from Millipore Sigma (i.e. Sigma-Aldrich Product W433400, pentadecanoic acid 99%; Product H3500, heptadecanoic acid, ≥ 98%).

### Cell-based peroxisome proliferator-activated receptor (PPAR) agonist assays

Based on known PPAR ligand and agonist activities of polyunsaturated fatty acids, it was hypothesized that C15:0, as a fatty acid, may also be a PPAR ligand. Chinese hamster ovary (CHO, cell line 93-0457C2) cell-based functional protein interaction assays for PPARα, δ, and γ that are part of the Eurofins/DiscoverX PathHunter NHRscan panel (Fremont, CA) were used to evaluate C15:0 PPARα, δ and γ agonist activities at 10 concentrations (up to 100 µM). Effective concentrations (EC50) and maximum activities were determined in relation to positive controls (GW7647 for PPARα, L-165,041 for PPARδ, and troglitazone for PPARγ). Detailed methods used for these assays and assay readouts are available from Eurofins/DiscoverX.

### Off-target pharmacology

It was hypothesized that C15:0 would not have off-target pharmacology. To test this hypothesis, the Eurofins/DiscoverX SafetyScan47 panel was used to test C15:0 at 10 concentrations for agonist and antagonist activities across 76 assays and 47 genes and targets that are relevant to compound safety and mechanisms of action, including G-protein coupled receptors, kinases, transporters, ion channels, nuclear receptors, and non-kinase enzymes (Supplement Table 1). Detailed methods used for these assays and assay readouts are available from Eurofins/DiscoverX.

### Mitochondrial reactive oxygen species (ROS) assays

We developed an assay in-house to evaluate the effect of increasing concentrations of C15:0 (10, 20, 50, 100 and 200 µM) on stressed HepG2 cell systems with dysfunctional mitochondria producing ROS. The basis of our assay was triphenylphosphonium dihydroethidium, which allowed for electrophoretically driven uptake of the probe in actively respiring mitochondria (unmodified dihydroethidium is limited to the cytosol). The dihydroethidium moiety reacts with superoxide and generates a fluorescent signal by hydroxylating the 2 position, generating fluorescent hydroxyethidium. This allowed detection of ROS in the mitochondria of live cells. HepG2 human liver carcinoma cells were cultured in High Glucose Dulbecco’s modified eagles medium (DMEM Gibco 11965092) with 10% fetal bovine serum (FBS). Cells were passed every 3 days using TrypLE disassociation reagent and grown at 37 °C with 5% CO_2_ and 100% humidity. Seed cells were grown in T75 flasks. Assay cells were grown in T25 flasks.

Incubation of cells under various conditions were started 18–24 hours before the assay. T25 flasks were aspirated of high glu media, washed with 7 ml’s DPBS (Gibco 14040-133) and the media replaced with 7 ml’s of Substrate Limited media supplemented with 5% FBS and 20% of appropriate test condition. The typical final concentration was 33 μM BSA and 200 μM FFA as per Agilent protocol for a 6:1 FFA:BSA ratio. HepG2 cells in T25 flasks were aspirated of media, washed with 7 ml of Dulbecco’s phosphate buffered saline (DPBS) and aspirated to remove wash. 2 ml’s of TrypLE was added to the flask and placed back in a humidified incubator for 5–10 minutes until cells were fully detached. Cells were transferred to a 15 ml conical tube and the flask was washed with an additional 2 ml’s of DPBS which was also added to the 15 ml conical tube. Cells were spun at 140 RCF for 5 minutes and supernatant was decanted. 5 ml’s of DMEM (Substrate Limited media + 10% 170 μM BSA solution) was added to cell pellet and cells were re-suspended by pipette.

Re-suspended cells were diluted to ~ 2.0 ×10^6^ cells/ml in serum starved DMEM (Substrate Limited Media + 20% test solution) and 300ul was plated into each well of a 96 well conical polypropylene plate (Nunc 339651). Cells were incubated for 1 hr at 37 °C with gentle agitation. After incubation, the cells were spun at 300 RCF for 5 minutes and decanted. 300ul of 2uM MitoSOX red in DPBS was added to each well except for blanks. Cells were incubated for 30 minutes at 37 °C with gentle agitation. Cells were spun at 300 RCF for 5 minutes and decanted. Cells were washed (3×) with 300ul of warmed (37 °C) DPBS, re-suspended by pipette and spun at 300 RCF for 5 minutes, then decanted and the procedure repeated. Cells were re-suspended by pipette during each wash. After final wash, cells were re-suspended in another 300ul of DPBS. Re-suspended cells were then transferred to 1.5 ml Eppendorf tubes for analysis on Attune NxT flow cytometer. Cells were analyzed by flow cytometry with the following parameters. FSC = 8, SSC = 300, BL2 (corresponding to ~510ex/580em) =400, 100 ul/min flow rate, 150 ul total volume. Raw FCS files were analyzed by FlowJo (v10.5.0) to determine the percent ROS positive cells within those cells determined to be live and singlets (FSCH vs FSCA ~ 1). Seven replicate measurements were made for every concentration. Wilcoxon tests and two-sided P values were used to compare percent mitochondrial ROS between each C15:0 supplemented group (by concentration) and the non-supplemented control group. To evaluate Multiple saturated fatty acids (C13:0, C14:0, C15:0, C16:0, and C17:0) at 20 µM were then assessed for reduction of mitochondrial ROS. Due to low sample sizes and inability to assume normal distributions, Kruskall Wallis tests were used to evaluate differences in percent mitochondrial ROS among the different groups. Significance for all studies was defined as a two-sided P value ≤ 0.05.

### Human cell phenotypic profiling

It was hypothesized that, given its role as a natural PPARα/δ ligand, C15:0 would have anti-inflammatory and antifibrotic activities in human cell systems and that these activities would be better than other saturated fatty acids (C13:0, C14:0, C16:0 and C17:0). To test this hypothesis, primary human cell systems from the BioMAP Diversity PLUS panel (Eurofins/DiscoverX, Fremont, CA) mimicking inflammation and fibrosis were exposed to C15:0 at four concentrations (740 nm and 2.2, 6.7 and 20 µM). Quantitative measurements of 148 biomarker activities across this broad panel, along with comparative analysis of biological activities from known bioactive agents, were used to predict and compare the efficacy and function of C15:0 across these systems compared to non-supplemented control systems. Activated BioMAP systems were incubated with C15:0 for 24 to 72 hours. Biomarker activities were annotated when 2 or more consecutive concentrations changed in the same direction relative to at least six vehicle controls, were outside of the 95^th^ percentile significance envelope and had at least one concentration with an effect size> 20% (log10 ratio)> 0.1. Three cell systems (SAg, BT and HDF3CGF) were the focus of our study due to detected annotated activities by C15:0. The SAg system mimics chronic inflammation and autoimmune disease. In this system, human primary venular endothelial cells and peripheral blood mononuclear cells were exposed to TCR ligands (1×) to model Th1 type chronic inflammation. The BT system mimics asthma, oncology, autoimmunity and allergy. In this system, human primary peripheral blood mononuclear cells and B cells were exposed to α-IgM and TCR ligands (0.001×, sub-mitogenic levels) to model T cell dependent B cell proliferation. The HDF3CGF system mimics fibrosis and chronic inflammation. In this system, human primary dermal fibroblasts were exposed to IFNγ, TNFα, IL-1β, EGF, bFGF, and PDGF-BB to model fibrosis and Th1-type inflammation. Effects of C13:0, C14:0, C16:0 and C17:0 (20 µM) on these three cell systems were assessed and compared to C15:0.

### 24-hour pharmacokinetics study

Cell-based activity studies demonstrated C15:0 anti-inflammatory and antifibrotic activities between 6.7 and 20 µM. As such, a 24-hour pharmacokinetic study was conducted with 8- to 10-week old male Sprague Dawley rats (body weights ranging from 260 to 288 g) to evaluate if a single oral dose of C15:0 at 35 mg/kg body weight via gastric gavage could achieve 20 µM concentrations in the serum (equivalent to approximately 5.0 µg/ml). Briefly, six healthy rats were dosed once with deuterated C15:0 at 35 mg/kg orally in a methylcellulose suspension (10 mL/kg). The first 3 animals had blood collected pre-dose, and 15 min, and 1, 4, and 12 hrs post dosing. The final 3 animals had blood collected pre-dose, and 30 min, and 2, 8, and 24 hrs post dosing. Plasma deuterated C15:0 and C17:0 measurements were performed by the Genetics Laboratories at the Kennedy Krieger Institute. Fatty acids were analyzed by capillary gas chromatography/mass spectrometry of pentaflourobenzyl bromide fatty acid esters using an AT-Silar-100 column (Grace, Columbia, Maryland 21044) as previously described^79^.

### 14-day toxicology study

The purpose of this study was to determine the safety of daily oral pentadecanoic acid at higher doses over 14 days in 7- to 8-week old Sprague Dawley rats. There were four main study groups, with 5 animals/sex/group. The first three groups received pentadecanoic acid daily via gastric gavage for 14 days in a 10 mL/kg suspension: Group 1 = 35 mg/kg, Group 2 = 175 mg/kg, Group 3 = 350 mg/kg doses. Group 4 was the vehicle control group. Animals received the daily oral doses on Days 0–13. Once daily clinical observations were made during acclimation, and twice daily clinical observations were made during the dosing period. Weekly cage food consumption was measured. Body weights were measured pre-study (2 times), on Day 0, then weekly (5 body weights total). On Day 14, blood was collected and processed for clinical chemistries. Plasma total C15:0 and C17:0 measurements were performed by the Genetics Laboratories at the Kennedy Krieger Institute. Fatty acids were analyzed by capillary gas chromatography/mass spectrometry of pentaflourobenzyl bromide fatty acid esters using an AT-Silar-100 column (Grace, Columbia, Maryland 21044) as previously described^79^. At necropsy, liver, kidneys (combined), heart, and adrenal glands (combined) of animals were weighed and fixed in 10% neutral buffered formalin. Standard trimming, processing, slide preparation and histopathology of liver, kidney, heart, and adrenals from the vehicle (Group 4) and high dose (Group 3) groups were completed.

### Oral supplement study with in vivo obesity model

Forty 6-week old male C57BL/6J mice (Jackson Laboratories, Bar Harbor, ME) were fed a high fat diet (HFD, 60% kilocalories fat, D12492 Research Diets Inc., New Brunswick, NJ) for eight weeks. Animals were randomly assigned to one of the following four study groups (10 per group) on Day -1 based on body weight: Group 1 (HFD non-supplemented controls), Group 2 (daily oral 5 mg/kg heptadecanoic acid, C17:0), Group 3 (daily oral 35 mg/kg heptadecanoic acid, C17:0), and Group 4 (daily oral 5 mg/kg pentadecanoic acid, C15:0). Group sizes for this pilot study were based on numerous published studies initially evaluating the effects of other natural small molecules on similar metabolic indices in the same model. Animals were provided the vehicle (1x PBS) or test articles daily via gastric gavage for 12 weeks. Animals were fed the HFD throughout the study. The study was conducted by Charles River Laboratories (Shrewsbury, MA), where animals were housed singly in solid bottom polycarbonate cages with Beta-chip contact bedding and enviro-dri nesting material, with temperature and humidity ranges of 18° to 26 °C and 30 to 70%, respectively. A 12 hr light cycle was used, and water and food were provided ad libitum. The following blood-based variables were measured after 12 weeks of supplementation: glucose, insulin, triglycerides, cholesterol (total, HDL, LDL), IL-18, IL-6, monocyte chemoattractant protein 1 (MCP-1), plasminogen activation inhibitor 1 (PAI-1), tumor necrosis factor (TNF)α, free fatty acids, and ferritin. Body weights and body weight changes were recorded weekly throughout the study. Due to low sample sizes and inability to assume normal distributions, Wilcoxon tests were used to compare blood-based and body weight values between supplemented and non-supplemented controls; a two-sided P value ≤ 0.05 was defined as significant.

### Oral supplement study with *in vivo* NASH model

Sixteen male New Zealand white rabbits, 12- to 13- weeks old and weighing approximately 3 kg upon ordering (Charles River Canada from Kitayama Labs K.K. of Nagano Prefecture, Japan) were fed a high fat, high cholesterol diet (HFHC diet, LabDiet 5321, 4% peanut oil, 0.5% cholesterol, 2% maltose dextrin; Dyet Product #621098, Bethlehem, PA) for two weeks. Animals were randomly assigned to one of the following two study groups (8 per group) on Day -1 based on body weight: Group 1 (HFHC diet supplemented controls) and Group 2 (HFHC diet supplemented with 0.07% pentadecanoic acid; fed to achieve 35 mg/kg body weight daily oral dosing). Animal group numbers for this study were based on a power analysis on data from the prior mouse study; using a type-I error rate of 0.05 (alpha) and 80% power for two independent study groups with a continuous endpoint, the minimum sample size per group was six. An additional negative control, low fat diet group (Group 3, LabDiet #5321) with 8 animals was included. Animals in Groups 1 and 2 were fed the HFHC diet throughout the supplement period (11 weeks). The study was conducted by PreClinical Research Services (Fort Collins, CO), where animals were housed singly in steel or plastic cages, with temperature ranges of 16° to 22 °C. A 12 hr light cycle was used, and water was provided ad libitum. Complete blood cell count, red blood cell indices, clinical chemistries, and iron panels were measured in blood samples collected from animals after 11 weeks of supplementation. Liver histology scoring, including fibrosis, hepatocyte ballooning, lobular inflammation, microgranuloma, steatosis, and iron deposition, was conducted by Charles River Pathology Services (Frederick, MD). Due to low sample sizes and inability to assume normal distributions, Wilcoxon tests were used to compare blood-based and liver histology values between supplemented and non-supplemented controls; a two-sided P value ≤ 0.05 was defined as significant.

## Supplementary information

Supplementary information.

## Data Availability

The datasets generated during and/or analyzed as part of the current study are either provided as Supplementary Materials or are available from the corresponding author on reasonable request.
