# Supplementary material for: Efficacy of dietary odd-chain saturated fatty acid pentadecanoic acid parallels broad associated health benefits in humans: could it be essential?
Source: Sci Rep. 2020 May 18;10:8161. doi: 10.1038/s41598-020-64960-y (PMC7235264; doi:10.1038/s41598-020-64960-y)
Supplement: Supplementary file 1 — Supplementary information. [file 41598_2020_64960_MOESM1_ESM.pdf]

SUPPLEMENTARY INFORMATION

Efficacy of dietary odd-chain saturated fatty acid pentadecanoic acid parallels broad associated health benefits in humans: could it be essential?

Stephanie Venn-Watson[1,2]*, Richard Lumpkin[2], Edward A. Dennis[3]

[1]Seraphina Therapeutics, Inc. San Diego, CA 92106

[2] Epitracker Inc., San Diego, CA 92106

[3]Department of Chemistry and Biochemistry and Department of Pharmacology, University of California at San Diego, La Jolla, CA 92093-0601

Supplemental Tables and Figures

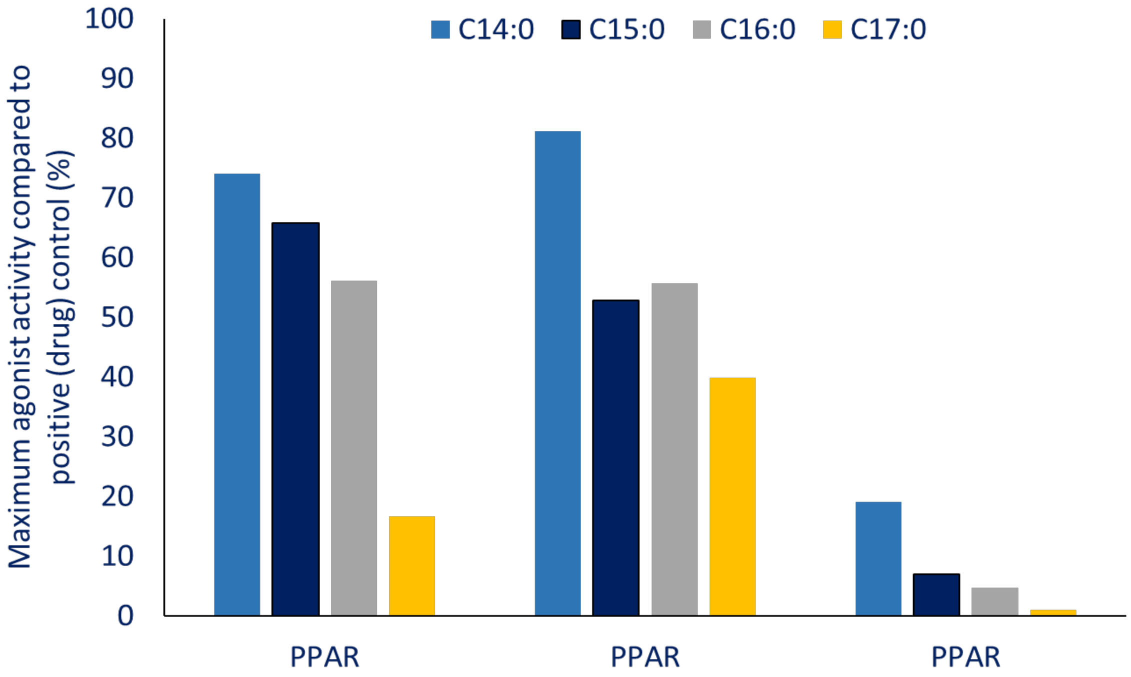

Supplement Fig 1. Maximum peroxisome proliferator-activated receptor (PPAR) agonist activities of saturated fatty acids compared to positive controls GW7647, L-165,041 and troglitazone for PPARα, PPARδ and PPARγ, respectively.

Supplement Table 1. Use of cell-based assays to evaluate potential off-target pharmacological activities of pentadecanoic acid (C15:0, > 99%), including agonist and antagonist activities relevant to compound safety.

Target Class	Assay Name	Assay Target	Mode	Result Type	Value prefix	RC (μM)
GPCR	Calcium Flux	ADORA2A	Agonist	EC50	>	20
GPCR	Calcium Flux	ADRA1A	Agonist	EC50	>	20
GPCR	Calcium Flux	AVPR1A	Agonist	EC50	>	20
GPCR	Calcium Flux	CCKAR	Agonist	EC50	>	20
GPCR	Calcium Flux	CHRM1	Agonist	EC50	>	20
GPCR	Calcium Flux	CHRM3	Agonist	EC50	>	20
GPCR	Calcium Flux	EDNRA	Agonist	EC50	>	20
GPCR	Calcium Flux	HRH1	Agonist	EC50	>	20
GPCR	Calcium Flux	HTR2A	Agonist	EC50	>	20
GPCR	Calcium Flux	HTR2B	Agonist	EC50	>	20
GPCR	Calcium Flux	ADORA2A	Antagonist	IC50	>	20
GPCR	Calcium Flux	ADRA1A	Antagonist	IC50	>	20
GPCR	Calcium Flux	AVPR1A	Antagonist	IC50	>	20
GPCR	Calcium Flux	CCKAR	Antagonist	IC50	>	20
GPCR	Calcium Flux	CHRM1	Antagonist	IC50	>	20
GPCR	Calcium Flux	CHRM3	Antagonist	IC50	>	20
GPCR	Calcium Flux	EDNRA	Antagonist	IC50	>	20
GPCR	Calcium Flux	HRH1	Antagonist	IC50	>	20
GPCR	Calcium Flux	HTR2A	Antagonist	IC50	>	20
GPCR	Calcium Flux	HTR2B	Antagonist	IC50	>	20
GPCR	cAMP	ADRA2A	Agonist	EC50	>	20
GPCR	cAMP	ADRB1	Agonist	EC50	>	20
GPCR	cAMP	ADRB2	Agonist	EC50	>	20
GPCR	cAMP	CHRM2	Agonist	EC50	>	20
GPCR	cAMP	CNR1	Agonist	EC50	>	20
GPCR	cAMP	CNR2	Agonist	EC50	>	20
GPCR	cAMP	DRD1	Agonist	EC50	>	20
GPCR	cAMP	DRD2S	Agonist	EC50	>	20
GPCR	cAMP	HRH2	Agonist	EC50	>	20
GPCR	cAMP	HTR1A	Agonist	EC50	>	20
GPCR	cAMP	HTR1B	Agonist	EC50	>	20
GPCR	cAMP	OPRD1	Agonist	EC50	>	20
GPCR	cAMP	OPRK1	Agonist	EC50	>	20
GPCR	cAMP	OPRM1	Agonist	EC50	>	20
GPCR	cAMP	ADRA2A	Antagonist	IC50	>	20

GPCR	cAMP	ADRB1	Antagonist	IC50	>	20
GPCR	cAMP	ADRB2	Antagonist	IC50	>	20
GPCR	cAMP	CHRM2	Antagonist	IC50	>	20
GPCR	cAMP	CNR1	Antagonist	IC50	>	20
GPCR	cAMP	CNR2	Antagonist	IC50	>	20
GPCR	cAMP	DRD1	Antagonist	IC50	>	20
GPCR	cAMP	DRD2S	Antagonist	IC50	>	20
GPCR	cAMP	HRH2	Antagonist	IC50	>	20
GPCR	cAMP	HTR1A	Antagonist	IC50	>	20
GPCR	cAMP	HTR1B	Antagonist	IC50	>	20
GPCR	cAMP	OPRD1	Antagonist	IC50	>	20
GPCR	cAMP	OPRK1	Antagonist	IC50	>	20
GPCR	cAMP	OPRM1	Antagonist	IC50	>	20
Ion Channel	Ion Channel	CAV1.2	Blocker	IC50	>	20
Ion Channel	Ion Channel	GABAA	Blocker	IC50	>	20
Ion Channel	Ion Channel	hERG	Blocker	IC50	>	20
Ion Channel	Ion Channel	HTR3A	Blocker	IC50	>	20
Ion Channel	Ion Channel	KvLQT1/minK	Blocker	IC50	>	20
Ion Channel	Ion Channel	nAChR(a4/b2)	Blocker	IC50	>	20
Ion Channel	Ion Channel	NAV1.5	Blocker	IC50	>	20
Ion Channel	Ion Channel	NMDAR (1A/2B)	Blocker	IC50	>	20
Ion Channel	Ion Channel	GABAA	Opener	EC50	>	20
Ion Channel	Ion Channel	HTR3A	Opener	EC50	>	20
Ion Channel	Ion Channel	KvLQT1/minK	Opener	EC50	>	20
Ion Channel	Ion Channel	nAChR(a4/b2)	Opener	EC50	>	20
Ion Channel	Ion Channel	NMDAR (1A/2B)	Opener	EC50	>	20
Kinases	Binding	INSR	Inhibitor	IC50	>	20
Kinases	Binding	LCK	Inhibitor	IC50	>	20
Kinases	Binding	ROCK1	Inhibitor	IC50	>	20
Kinases	Binding	VEGFR2	Inhibitor	IC50	>	20
NHR	NHR Nuclear Translocation	AR	Agonist	EC50	>	20
NHR	NHR Nuclear Translocation	AR	Antagonist	IC50	>	20
NHR	NHR Protein Interaction	GR	Agonist	EC50	>	20
NHR	NHR Protein Interaction	GR	Antagonist	IC50	>	20
Non-Kinase Enzymes	Enzymatic	AChE	Inhibitor	IC50	>	20
Non-Kinase Enzymes	Enzymatic	COX1	Inhibitor	IC50	>	20
Non-Kinase Enzymes	Enzymatic	COX2	Inhibitor	IC50	>	20
Non-Kinase Enzymes	Enzymatic	MAOA	Inhibitor	IC50	>	20
Non-Kinase Enzymes	Enzymatic	PDE3A	Inhibitor	IC50	>	20

Non-Kinase Enzymes	Enzymatic	PDE4D2	Inhibitor	IC50	>	20
Transporter	Transporter	DAT	Blocker	IC50	>	20
Transporter	Transporter	NET	Blocker	IC50	>	20
Transporter	Transporter	SERT	Blocker	IC50	>	20

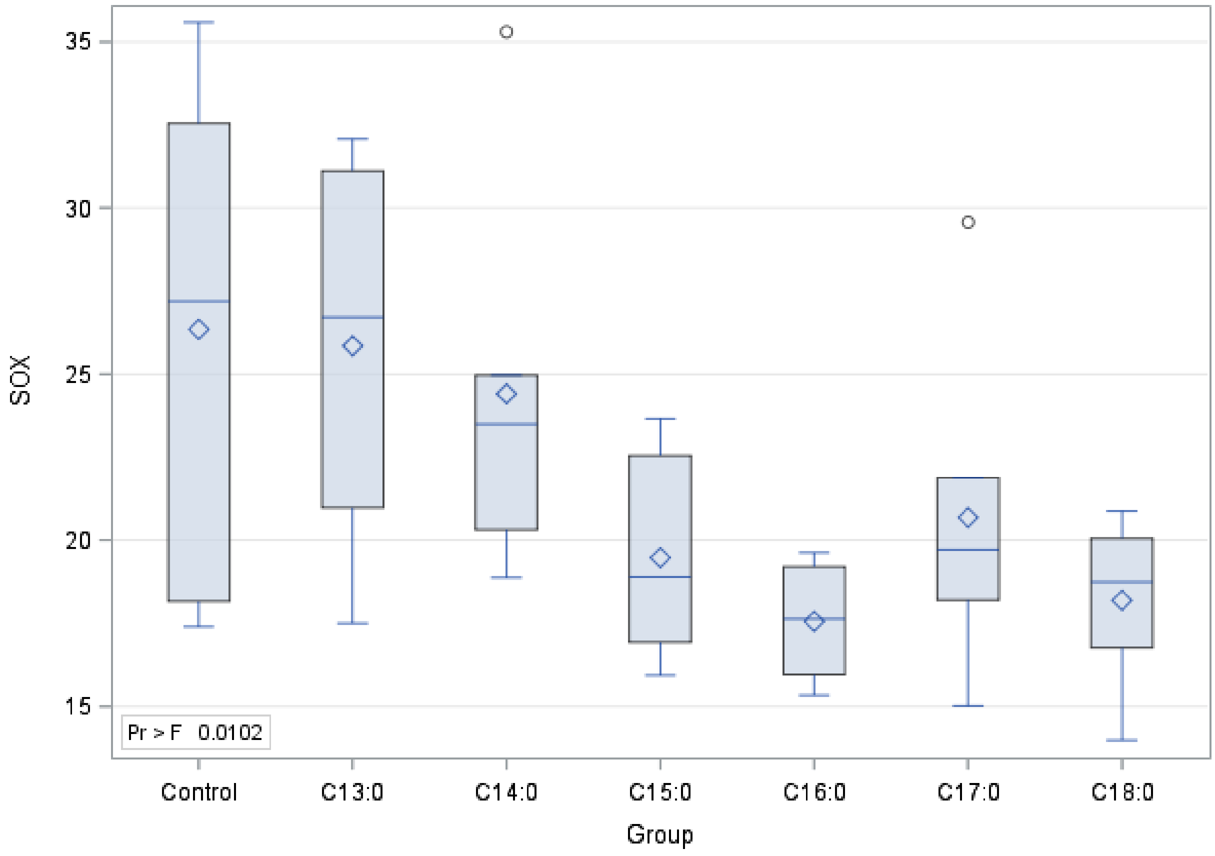

Supplement Fig 2. Decreased percent cells with mitochondrial reactive oxygen species (SOX) production in stressed human cell systems treated with different saturated fatty acids at 20 µM compared to non-treated controls (n=7 runs per concentration). Endpoint of upper whisker, maximum; upper edge of box, 75th percentile; line inside box, median; diamond, mean; lower edge of box, 25th percentile; endpoint of lower whisker, minimum; circle, outlier.

Supplement Table 2. Evaluation of potential cytotoxicity of C15:0 at four concentrations with a variety of primary human cell systems. Cytotoxicity was defined as a decrease in protein content, measured by Sulforhdamin B (SRB) staining, of 50% (Log_{10} of -0.3) or greater than non-exposed control cell systems. Primary human cell systems names are based on the Eurofins Discovery BioMAP Diversity PLUS platform.

Primary Human Cell System	Primary Human Cell Type	Cytotoxicity Measurement	Log-transformed ratio of cytotoxicity biomarker readout for C15:0-treated sample (n=1) over vehicle controls (n ≥ 6)				Cytotoxic Concentrations
			C15:0, 20 uM	C15:0 6.7 uM	C15:0, 2.2 uM	C15:0, 0.74 uM	
3C	Venular endothelial cells	SRB	-0.00165976	0.009775274	0.004730171	0.053479757	None
4H	Venular endothelial cells	SRB	0.029839156	0.008110998	-0.004865047	0.006280846	None
LPS	Venular endothelial cells and peripheral blood mononuclear cells	SRB	-0.009512417	-0.005624347	0.000525058	0.000525058	None
SAg	Venular endothelial cells	SRB	-0.022253469	0.030201286	0.014647746	-0.008889508	None
BT	Primary blood mononuclear cells	SRB	0.03712544	0.014101566	0.010687284	-0.009393062	None
BF4T	Bronchial epithelial cells and dermal fibroblasts	SRB	0.00198258	0.00693601	-0.017189393	0.021185795	None
BE3C	Bronchial epithelial cells	SRB	3.84485E-05	-0.031531524	-0.013710151	-0.000577136	None
CASM3C	Coronary artery smooth muscle cells	SRB	0.008939704	0.01807596	0.034825876	-0.00319706	None
HDF3CGF	Dermal fibroblasts	SRB	-0.019816725	0.007378762	0.010495189	-0.01414577	None
KF3CT	Keratinocytes and dermal fibroblasts	SRB	-0.003082849	-0.010100327	0.001915727	-0.00579841	None
MyoF	Lung fibroblasts	SRB	-0.018901613	-0.002103599	-0.008426469	0.017832216	None
lMphg	Venular endothelial cells and macrophages	SRB	0.01315957	-0.00239397	-0.03165984	0.027546791	None
lMphg	Macrophages alone	SRB	-0.058689274	-0.037816864	0.000725812	0.023726808	None

Supplement Table 3. Comparisons of indices among Sprague-Dawley rats supplemented with once daily, oral pentadecanoic acid (C15:0, > 98%) (35, 175, and 350 mg/kg body weight) for 14 days with non-supplemented vehicle controls. p value = compared to control group. *Similar minimal to mild changes detected in both supplemented and control groups.

Safety index (Day 14)	Normal range	Vehicle controls (n=10)	Low dose C15:0 (35 mg/kg) (n=10)		Moderate dose C15:0 (175 mg/kg) (n=10)		High dose C15:0 (350 mg/kg) (n=10)	
		No.	No.	p value	No.	p value	No.	p value
**Abnormal clinical chemistries (no.)**								
Albumin	3.2 - 4.4	1	0	0.5	0	0.5	0	0.5
Alkaline phosphatase	134-588	0	1	0.5	0	1.0	0	1.0
ALT	37-178	0	1	0.5	1	0.5	0	1.0
AST	64-386	0	1	0.5	0	1.0	0	1.0
BUN	7-23	3	0	0.1	1	0.3	1	0.3
Calcium	10.3-14.3	0	0	1.0	0	1.0	1	0.5
Cholesterol	64-158	1	0	0.5	0	0.5	0	0.5
GGT	0-7	5	2	0.2	3	0.2	2	0.2
Glucose	110-456	1	1	0.5	1	0.5	0	0.5
Potassium	5.98-11.90	2	1	0.4	0	0.2	0	0.2
Sodium	138.3-171.3	0	0	1.00	0	1.0	0	1.0
Phosphorous	7.9-16.6	3	2	0.4	4	0.3	2	0.4
Protein	6.0-8.2	1	0	0.5	0	0.5	0	0.5
**Total plasma fatty acids (µg/ml)**		Mean ± SD	Mean ± SD	p value	Mean ± SD	p value	Mean ± SD	p value
C15:0								
Females (n=5 / group)		3 ± 1	3 ± 1	0.27	4 ± 1	0.03	10 ± 5	0.02
Males (n=5 / group)		5 ± 1	7 ± 1	0.02	11 ± 4	0.02	14 ± 4	0.02
C17:0								
Females (n=5 / group)		6 ± 1	6 ± 1	0.42	7 ± 2	0.21	12 ± 2	0.02
Males (n=5 / group)		6 ± 1	7 ± 1	0.02	10 ± 2	0.02	11 ± 1	0.02
**Normal histology**								
Females (n=5 / group)			Not evaluated					
Adrenal glands		5					5	
Heart		5					5	
Kidneys		3*					2*	
Liver		1*					0*	
Males (n=5 / group)								
Adrenal glands		5					5	
Heart		5					3*	
Kidneys		0*					2*	
Liver		1*					0*	
**Body weights (g)**	p value	Mean ± SD	Mean ± SD		Mean ± SD		Mean ± SD	
Females (n=5 / group)	0.22	247 ± 12	250 ± 18		262 ± 6		260 ± 13	

Males (n=5 /group)	0.27	347 ± 21	362 ± 19	343 ± 7	344 ± 16
**Organ weight to body weight ratios**					
Females (n=5 / group)					
Adrenal glands		0.03 ± 0.01	0.03 ± 0.00	0.02 ± 0.00	0.03 ± 0.01
Heart		0.41 ± 0.05	0.47 ± 0.06	0.45 ± 0.07	0.47 ± 0.06
Kidneys		0.77 ± 0.05	0.75 ± 0.06	0.83 ± 0.06	0.75 ± 0.03
Liver		4.41 ± 0.65	4.37 ± 0.24	4.32 ± 0.32	4.39 ± 0.13
Males (n=5 / group)					
Adrenal glands		0.02 ± 0.00	0.02 ± 0.01	0.04 ± 0.06	0.02 ± 0.01
Heart		0.43 ± 0.06	0.43 ± 0.04	0.48 ± 0.08	0.46 ± 0.08
Kidneys		0.82 ± 0.04	0.84 ± 0.07	0.81 ± 0.09	0.81 ± 0.08
Liver		4.68 ± 0.14	4.92 ± 0.34	4.87 ± 0.41	4.97 ± 0.31

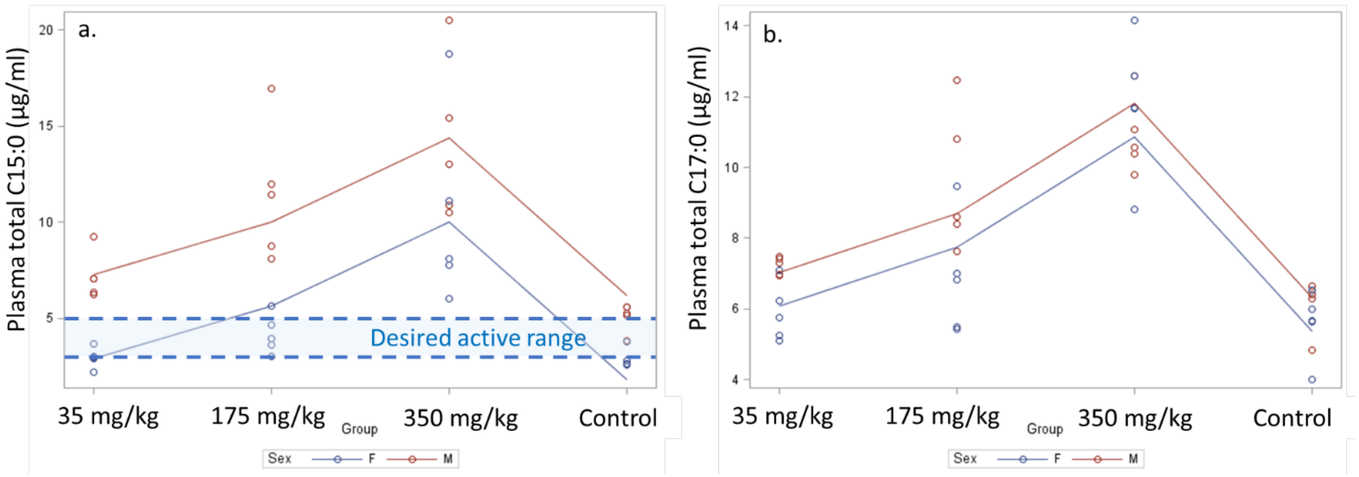

Supplement Fig 3. Plasma total C15:0 (a) and C17:0 (b) concentrations at Day 14 in Sprague Dawley rats (n=40 total, 5 females and 5 males per group) supplemented once daily with oral C15:0 (35, 175, and 350 mg/kg body weight).

Supplement Table 4. Comparisons of obesity-associated conditions between high fat diet-induced (HFD) obese C57BL/6J mice treated with daily oral odd-chain saturated fatty acids over 12 weeks and non-treated, diseased controls. *Wilcoxon two-sided P value ≤ 0.05 when comparing treatment group with non-treated HFD controls.

Health variable (Day 84)	Non-supplemented HFD controls (n=10)	Low-dose C17:0 (5 mg/kg) (n=10)	High-dose C17:0 (35 mg/kg) (n=10)	Low-dose C15:0 (5 mg/kg) (n=10)
Glucose (mg/dl)	307 ± 54	296 ± 23	282 ± 31	245 ± 37*
Insulin (uIU/ml)	12.2 ± 10.5	7.4 ± 4.8	6.8 ± 3.5	4.9 ± 3.8
Triglycerides (mg/dl)	84 ± 20	70 ± 10	68 ± 14	76 ± 20
Total cholesterol (mg/dl)	207 ± 20	197 ± 31	187 ± 44	183 ± 25*
IL-18 (pg/ml)	206 ± 84	187 ± 94	164 ± 82	144 ± 63
IL-6 (pg/ml)	60 ± 68	42 ± 40	38 ± 32	19 ± 11*
MCP-1 (pg/ml)	83 ± 31	86 ± 30	53 ± 32*	52 ± 25*
PAI-1 (pg/ml)	1.3 ± 0.4	1.4 ± 0.5	2.0 ± 0.9	2.2 ± 2.0
TNFα (pg/ml)	3.3 ± 2.1	3.7 ± 1.9	1.5 ± 1.5	1.7 ± 1.0
Free fatty acids (mmol/L)	1.0 ± 0.1	1.0 ± 0.1	1.0 ± 0.2	1.5 ± 1.3
Ferritin (ng/ml)	611 ± 204	586 ± 190	604 ± 127	531 ± 81
Body weight (g)	43 ± 7	43 ± 6	41 ± 7	41 ± 5
Body weight change (%)	132 ± 14	127 ± 9	122 ± 12	120 ± 6*

Supplement Table 5. Comparisons of indices among Sprague-Dawley rats supplemented with once daily, oral pentadecanoic acid (C15:0, > 98%) (35, 175, and 350 mg/kg body weight) for 14 days with non-supplemented vehicle controls. p value = compared to control group. *Similar minimal to mild changes detected in both supplemented and control groups.

	Serum concentrations (µg/ml)		
**Serum lipid species**	**Controls (n=4)**	**C15:0 dosed (5 mg/kg) (n=8)**	**p value**
C15:0 cholesterol ester	3.6 ± 0.4	2.9 ± 1.1	0.15
C15:0 diacylglycerol	0.06 ± 0.07	0.06 ± 0.07	0.50
C15:0 free fatty acid	7.1 ± 0.7	6.8 ± 1.8	0.40
C15:0 lysophosphatidylcholine	1.1 ± 0.1	1.1 ± 0.4	0.28
C15:0 lysophosphatidylethanolamine	0 ± 0	0.02 ± 0.02	0.05
C15:0 phosphatidylcholine	0.8 ± 0.4	1.4 ± 0.8	0.11
C15:0 triacylglycerol	2.4 ± 1.3	2.4 ± 1.1	0.40
C17:0 cholesterol ester	2.6 ± 0.1	2.0 ± 0.6	0.02
C17:0 free fatty acid	15 ± 1	13 ± 3	0.07
C17:0 lysophosphatidylethanolamine	0 ± 0	0.04 ± 0.01	0.03
C17:0 phosphatidylethanolamine	0.2 ± 0.4	0.2 ± 0.4	0.46
C17:0 triacylglycerol	3.9 ± 3.1	3.7 ± 2.3	0.47

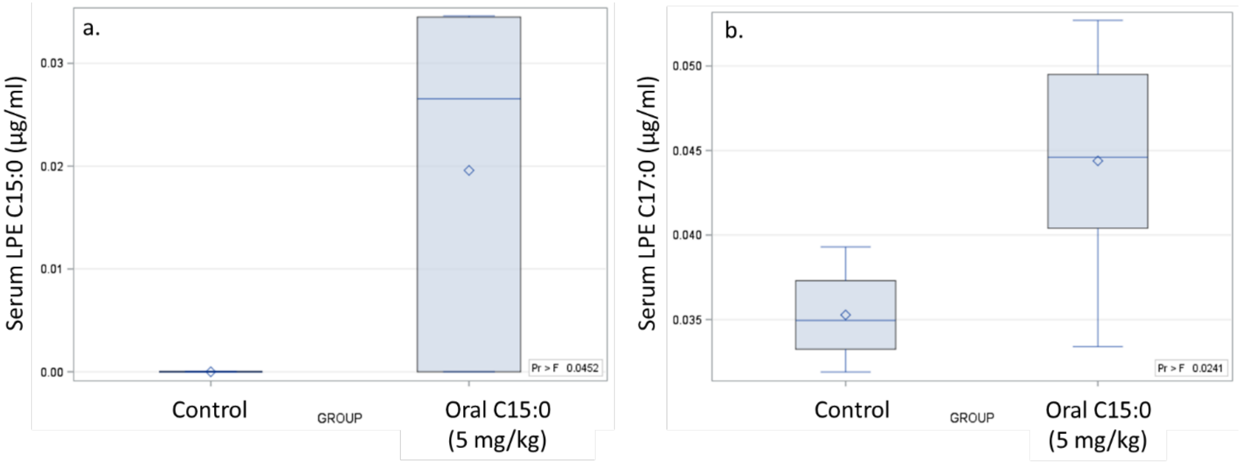

Supplement Fig 4. Serum lysophosphatidylethanolamide (LPE) C15:0 (a) and LPE C17:0 (b) concentrations at Day 90 in male C57BL/6J diet-induced obese mice treated once daily with oral C15:0 (5 mg/kg body weight, n=8) compared to controls (n=4).

Supplement Table 6. Comparisons of NASH-associated conditions between high fat, high cholesterol diet-induced (HFHC) NASH rabbits treated with daily oral C15:0 (35 mg/kg) over 11 weeks and non-treated, diseased controls. Wilcoxon two-sided P value ≤ 0.05*, ≤ 0.01** when comparing treatment group with non-treated HFHC diet controls. ^Wilcoxon two-sided P value ≤ 0.05 compared to standard diet controls.

**Health Variables** (Day 77)	**Standard diet controls (n=8)**	**Non-supplemented HFHC diet controls (n=8)**	**C15:0-supplemented HFHC diet group (n=8)**
**Liver Health**			
Blood-based indices			
Icterus (mg/dl)	0 ± 0	2.3 ± 2.8^	0.4 ± 0.5**
Total bilirubin (mg/dl)	0 ± 0	0.5 ± 0.3^	0.0 ± 0.1**
ALT (IU/L)	30 ± 9	23 ± 11	25 ± 11
AST (IU/L)	18 ± 9	34 ± 15^	26 ± 13
GGT (IU/L)	7 ± 1	132 ± 174^	240 ± 241^
Albumin (g/dl)	4.1 ± 0.5	3.2 ± 0.4^	3.8 ± 0.3**
Liver histology scores			
Fibrosis score	n/a	2.9 ± 0.4	2.3 ± 0.5*
Fibrosis pixel	n/a	3.0 ± 0.8	2.1 ± 0.8
Hepatocyte ballooning	n/a	1.0 ± 0	1.0 ± 0
Lobular inflammation	n/a	1.0 ± 0	1.1 ± 0.4
Microgranuloma	n/a	0.6 ± 0.5	0.5 ±0.5
Steatosis grade score	n/a	2.8 ± 0.7	3.0 ± 0
Steatosis location	n/a	1.0 ± 0	1.0 ± 0
Microvesicular steatosis	n/a	1.0 ± 0	1.0 ± 0
NAFLD score	n/a	4.8 ± 0.7	5.1 ± 0.4
**Iron Status**			
Blood-based indices			
Iron (µg/dl)	281 ± 38	229 ± 143^	200 ± 20^
Ferritin (ng/ml)	17 ± 19	11 ± 2	10 ± 2
UIBC (µg/dl)	0 ± 0	150 ± 180^	86 ± 49^
TIBC (µg/dl)	0 ± 0	195 ± 259	240 ± 133^
Transferrin saturation (%)	85 ± 1	65 ± 29	65 ± 11^
Liver histology			
Iron staining score (0-3)	n/a	1.1 ± 0.4	0.4 ± 0.5**
**Red Blood Cell Indices**			
Nucleated cells (10^3/µl)	4.1 ± 1.4	13 ± 9^	8 ± 1^*
Hemoglobin (g/dl)	15 ± 0.4	9 ± 2^	14 ± 1^**
Hematocrit (%)	46 ± 2	25 ± 5^	38 ± 4^**
Red blood cells (10^6/µl)	7.3 ± 0.3	3.5 ±1.2^	6 .0 ± 0.7^**
MCV (fl)	63 ± 2.3	79 ± 21^	64 ± 2**
MCH (pg)	21 ± 1	27 ± 5^	23 ± 1^
MCHC (g/dl)	33 ± 1	33 ± 4	36 ± 1^**
Red blood cell distribution width (%)	13 ± 1	21 ± 3^	15 ± 1^**
Reticulocytes (%)	2 ± 1	30 ± 22^	6 ± 2^**
Reticulocytes (actual)	181 ± 39	885 ± 497^	334 ± 71^**
**Lipids and Cardiovascular**			

Blood-based indices			
Total cholesterol (mg/dl)	23 ± 7	3576 ± 1018^	2516 ± 746^*
Triglycerides (mg/dl)	71 ± 38	755 ± 555^	235 ± 132^**
Platelets (10^3/µl)	335 ± 75	639 ± 123^	485 ± 87^*
MPV (fl)	6.3 ± 0.4	9.3 ± 2.3^	7.6 ± 0.6^
Other			
Mean arterial blood pressure	72 ± 15	57 ± 12^	74 ± 14*
**Inflammation**			
Globulins (g/dl)	1.9 ± 0.6	2.2 ± 0.3	1.1 ± 0.8**
C-reactive protein (ng/ml)	27 ± 10	71 ± 63^	40 ± 15^
**Other Health Indices**			
Glucose (mg/dl)	191 ± 19	124 ± 13^	145 ± 13^**
Insulin (uIU/ml)	3.7 ± 1.9	2.0 ± 0^	2.0 ± 0^
Creatine kinase (IU/L)	559 ± 168	2913 ± 2396^	731 ± 311**
Alkaline phosphatase (IU/L)	55 ± 15	53 ± 23	52 ± 16
Sodium (mEQ/L)	144 ± 1	132 ± 4^	136 ± 3^*
Chloride (mEQ/L)	102 ± 1	92 ± 4^	96 ± 1^*
Potassium (mEQ/L)	3.1 ± 0.4	3.2 ± 0.4	3.1 ± 0.3
Bicarbonate (mEQ/L)	20 ± 3	18 ± 3	20 ± 2
Anion gap (mmol/L)	25 ± 3	26 ± 4	24 ± 2
Osmolality (mOsm/kg)	292 ± 2	267 ± 7^	274 ± 5^
